# Supplementary material for: Genetic Factors That Could Affect Concussion Risk in Elite Rugby
Source: Sports (Basel). 2021 Jan 22;9(2):19. doi: 10.3390/sports9020019 (PMC7910946; doi:10.3390/sports9020019)
Supplement: Supplementary file 1 [file sports-09-00019-s001.pdf]

Supplementary Materials: Genetic Factors that Could Affect Concussion Risk in Elite Rugby

Mark R. Antrobus [1,2,*], Jon Brazier [1,3], Georgina K. Stebbings [1], Stephen H. Day [4], Shane M. Heffernan [5], Liam P. Kilduff [5], Robert M. Erskine [6,7] and Alun G. Williams [1,7]

[1] Sports Genomics Laboratory, Department of Sport and Exercise Sciences, Manchester Metropolitan University, Manchester M1 5GD, UK; j.brazier2@herts.ac.uk (J.B.); g.stebbings@mmu.ac.uk (G.K.S.); a.g.williams@mmu.ac.uk (A.G.W.)
[2] Sport and Exercise Science, University of Northampton, Northampton NN1 5PH, UK
[3] Department of Psychology and Sports Sciences, University of Hertfordshire, Hatfield AL10 9AB, UK
[4] Faculty of Science and Engineering, University of Wolverhampton, Wolverhampton WV1 1LY, UK; stephen.day@wlv.ac.uk
[5] Applied Sports, Technology, Exercise and Medicine (A-STEM) Research Centre, College of Engineering, Swansea University, Swansea SA1 8EN, UK; s.m.heffernan@swansea.ac.uk (S.M.H.); l.kilduff@swansea.ac.uk (L.P.K.)
[6] Research Institute for Sport & Exercise Sciences, Liverpool John Moores University, Liverpool L3 3AF, UK; r.m.erskine@ljmu.ac.uk
[7] Institute of Sport, Exercise and Health, University College London, London WC1E 6BT, UK
* Correspondence: mark.antrobus@northampton.ac.uk

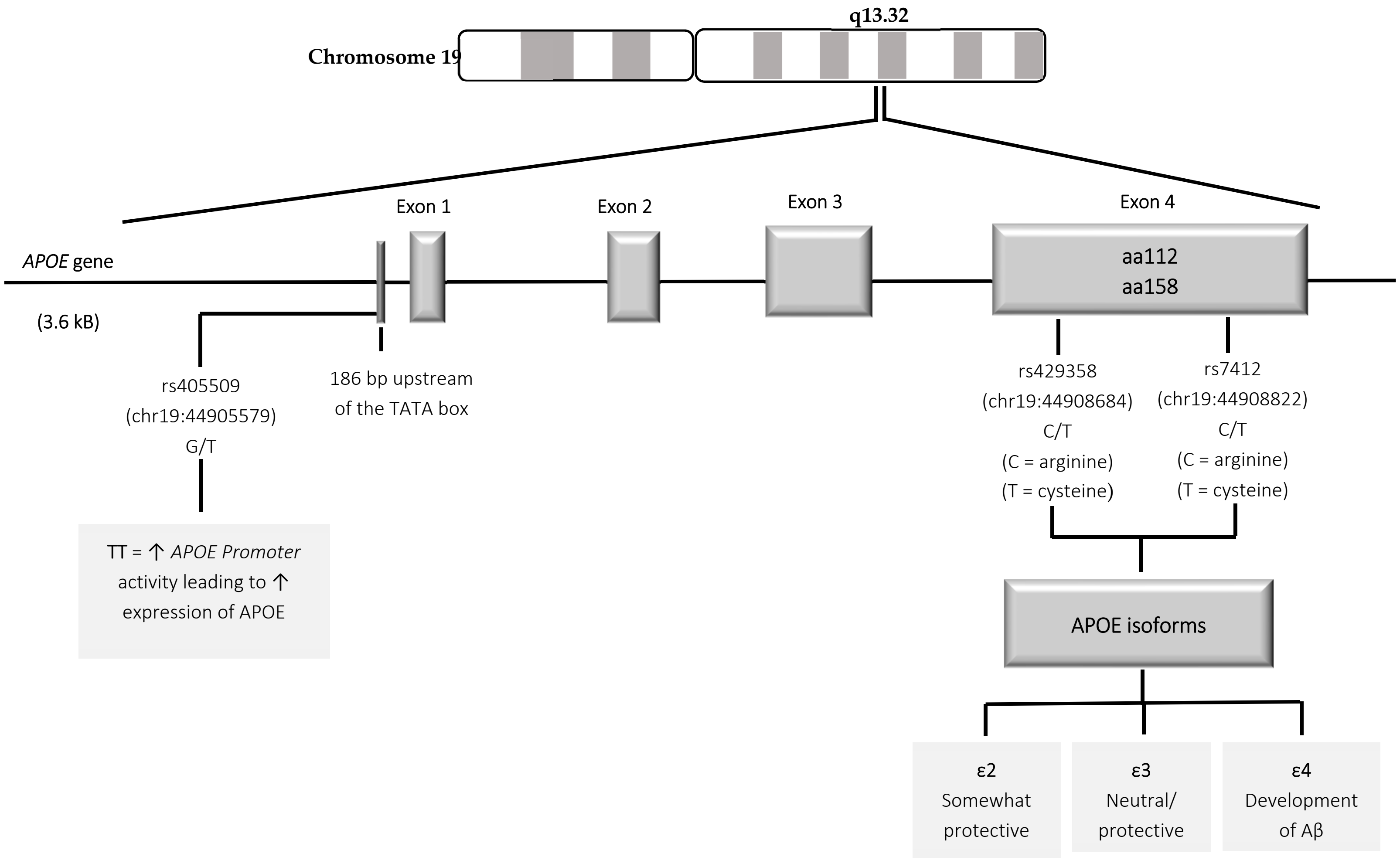

Figure S1. Schematic of *APOE* variants: The ε2 isoform binds with amyloid plaques (Aβ) and is removed enabling neuronal modelling and plasticity of neurons to be facilitated. The ε3 isoform binds with less infinity to Aβ, interacts with microtubules and is associated with neurite extension and branching. It also binds with tau to stabilise microtubules. The ε4 isoform does not bind with Aβ, meaning the activity of toxic cleaved APOE fragments can cause lysosomal leakage, leading to apoptosis and stimulation of tau and the formation of neurodegenerative neurofibrillary tangles

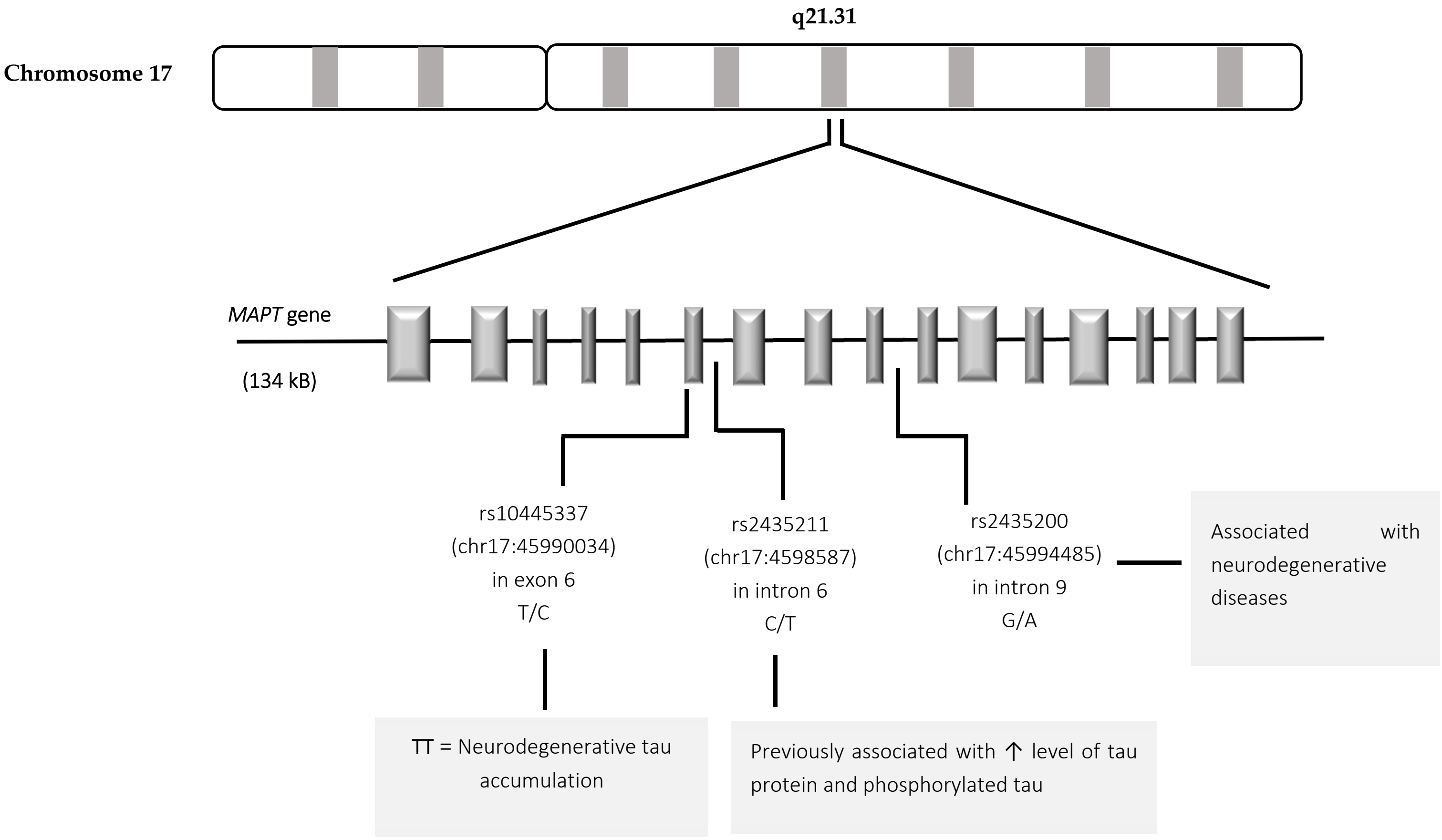

Figure S2. Schematic of *MAPT* polymorphisms associated with accumulation of tau clumps and the formation of neurofibrillary tangles and neuritic plaques.

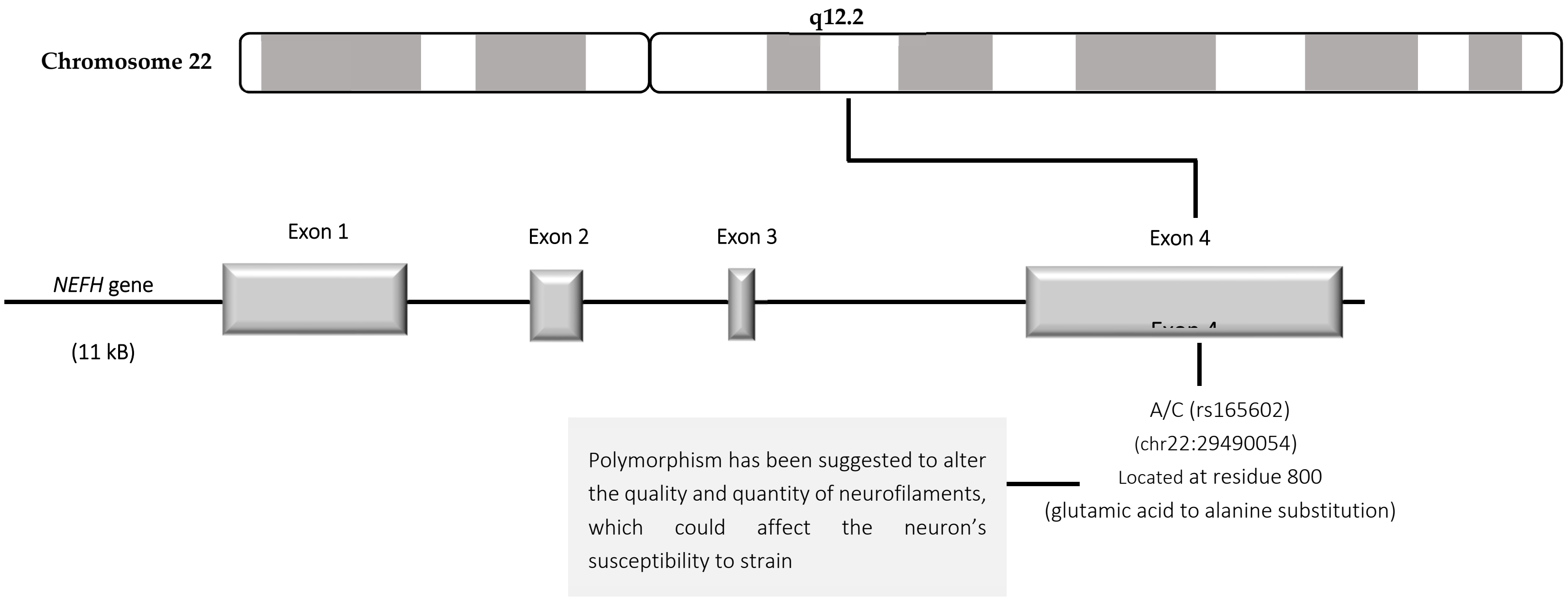

Figure S3. Schematic of rs165602 *NEFH* that encodes the heavy protein subunit of neurofilament, which combines with the medium and light subunits to form the neuronal cytoskeleton.

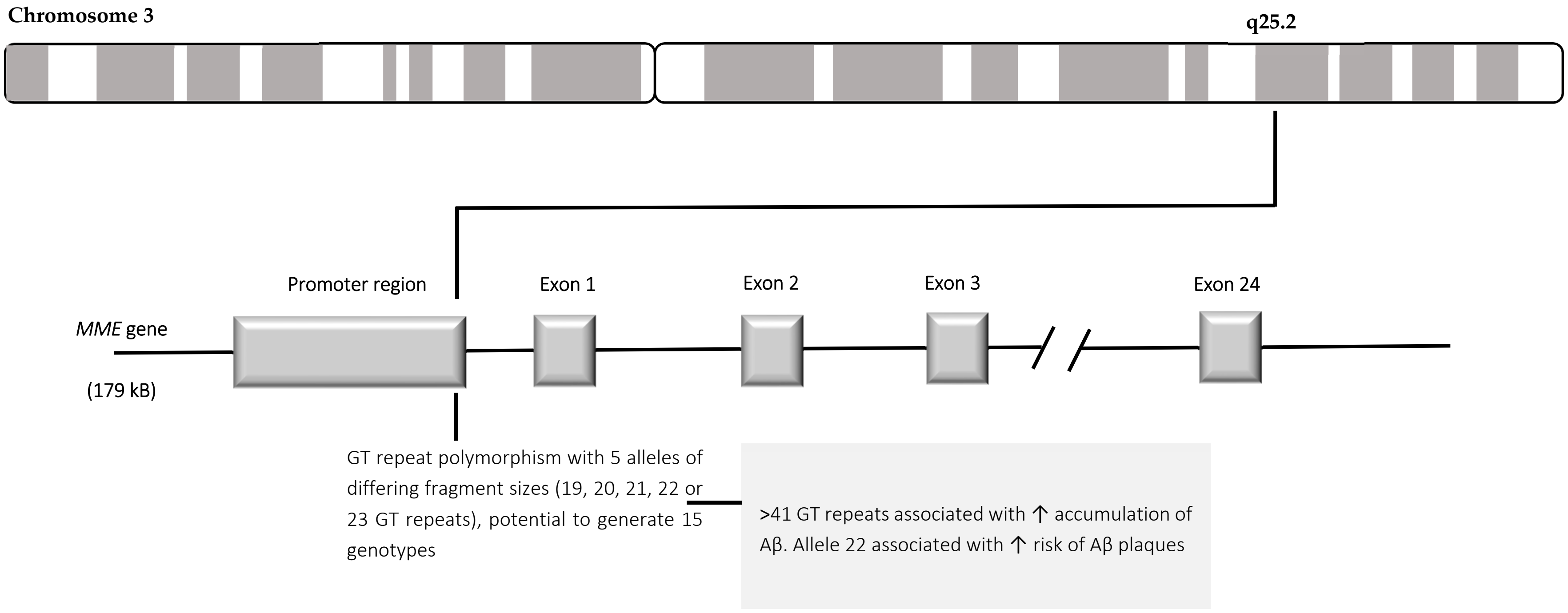

Figure S4. Schematic of GT repeat promoter polymorphism located in *MME* gene which encodes for neprilysin. Aβ, amyloid plaque.

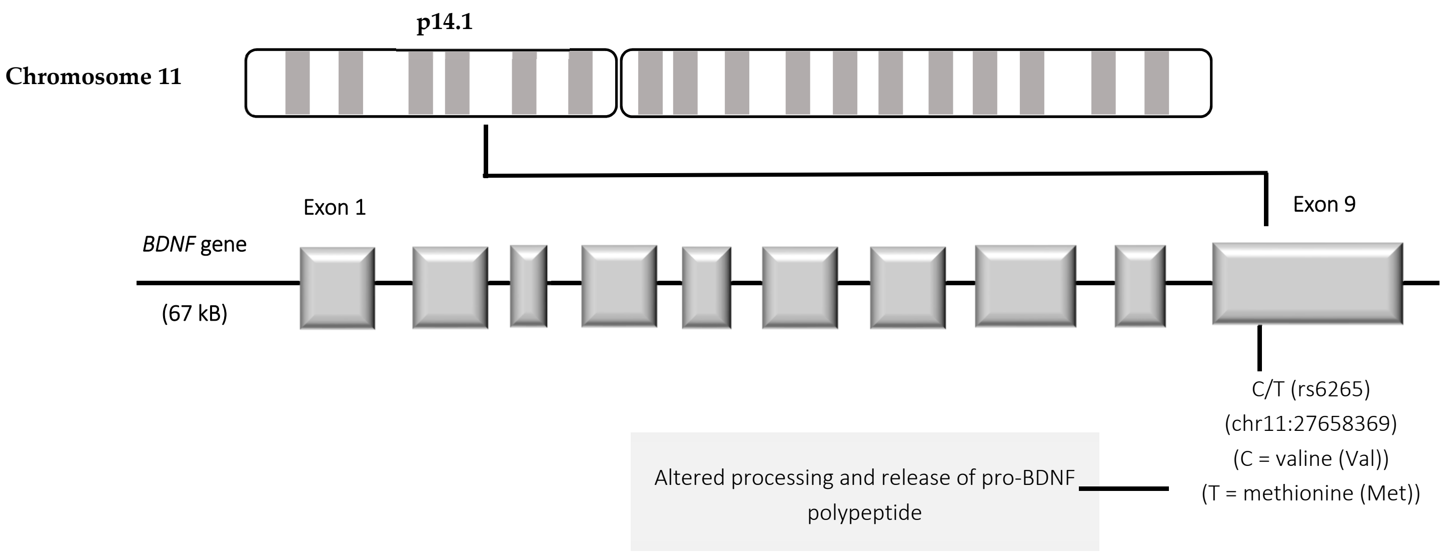

Figure S5. Schematic of *BDNF* (rs6265) Val to Met substitution results in poor packaging and intracellular protein trafficking of pro-BDNF polypeptide.

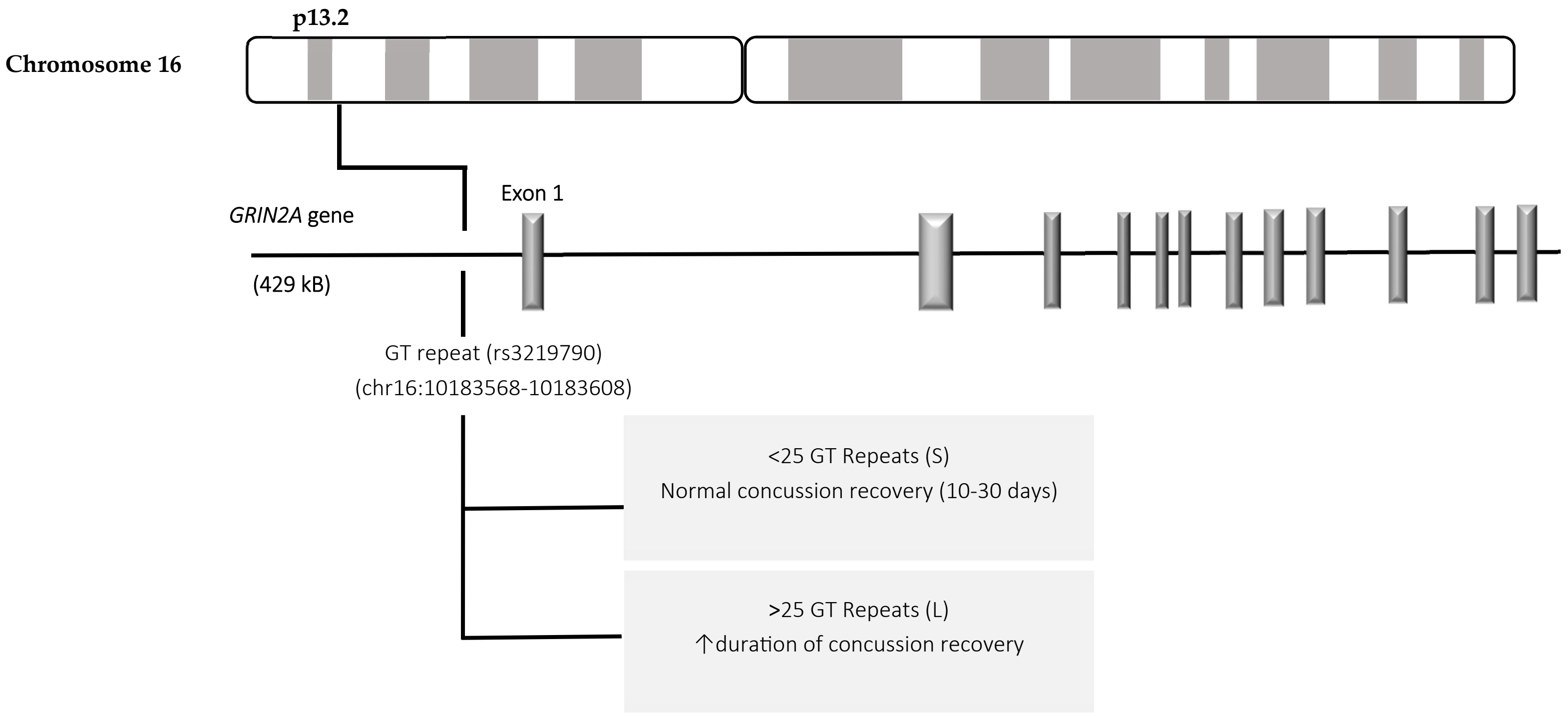

Figure S6. Schematic of *GRIN2A* promoter (rs3219790) the longer the GT repeat the lower the *GRIN2A* promoter activity.

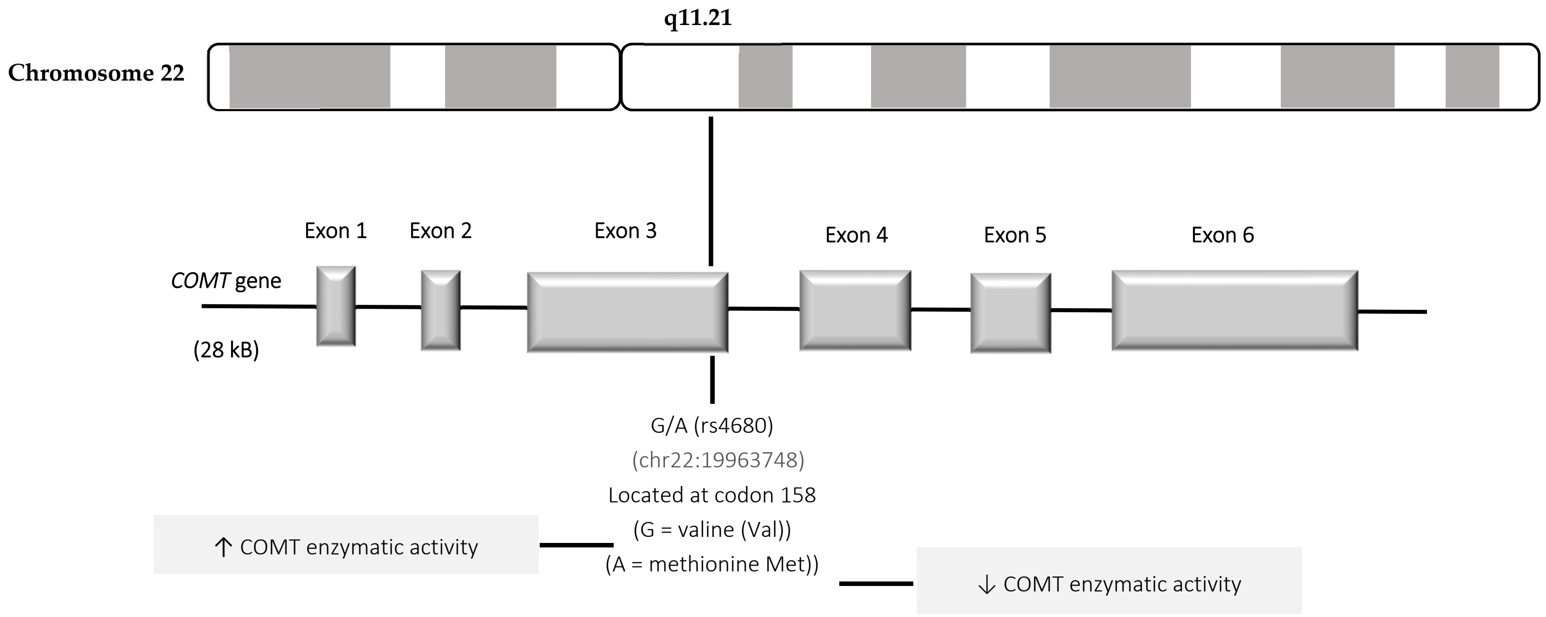

Figure S7. Schematic of rs4680 Catechol-O-methyltransferase.

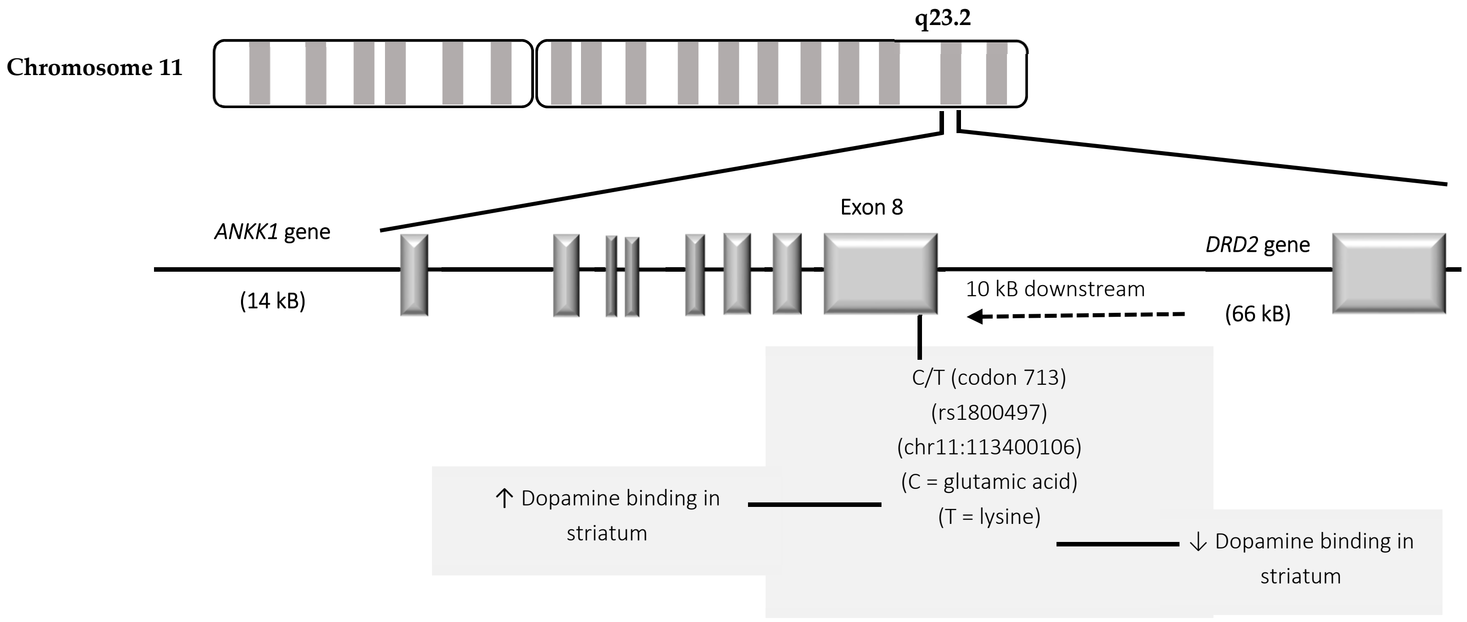

Figure S8. Schematic of *ankyrin repeat and kinase domain containing 1* (rs1800497). T allele of *ANKK1* has been associated with dopaminergic function.

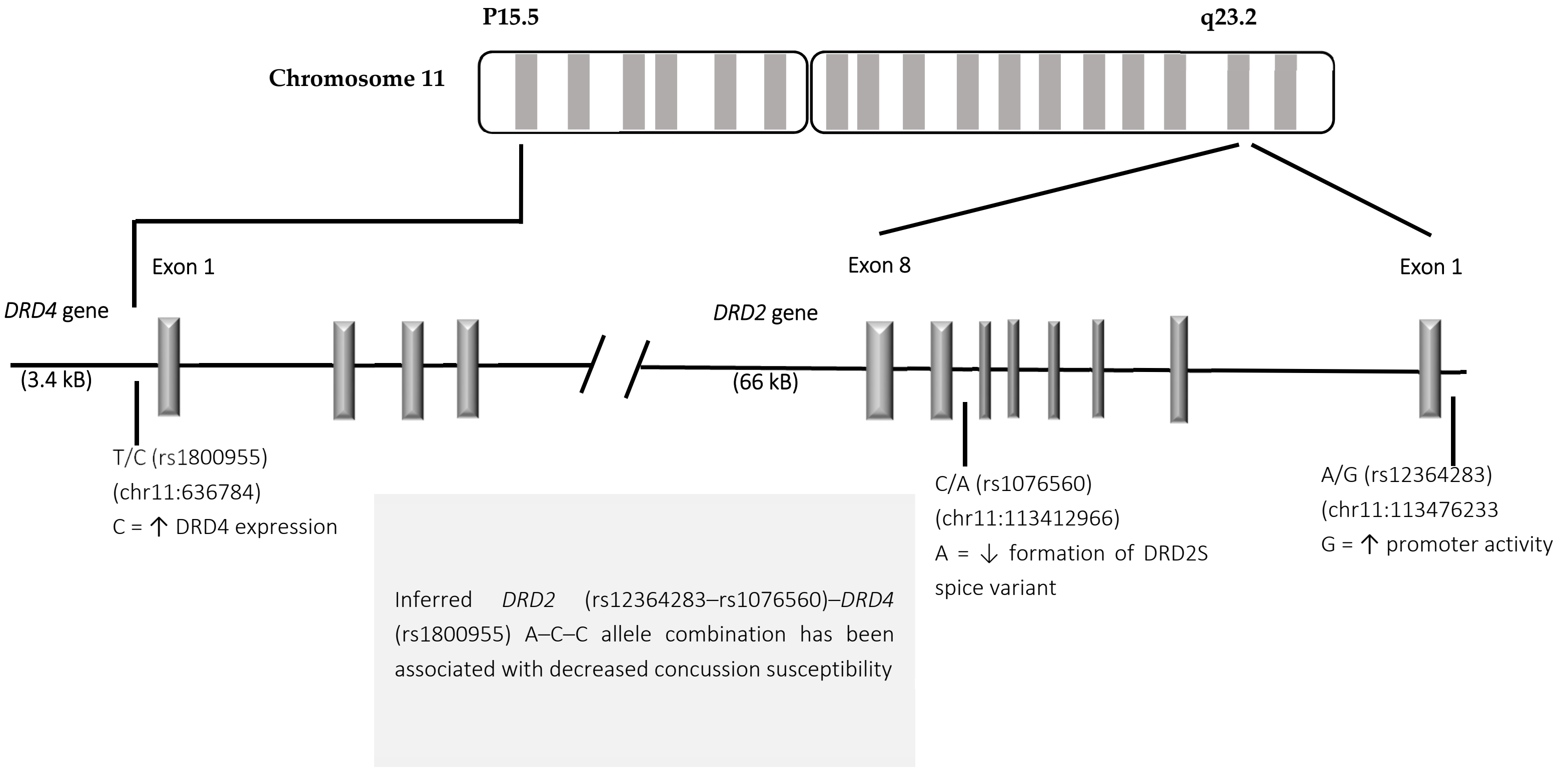

Figure S9. Schematic of *DRD2* (rs1076560 and rs12364283) *and DRD4* (rs1800955).

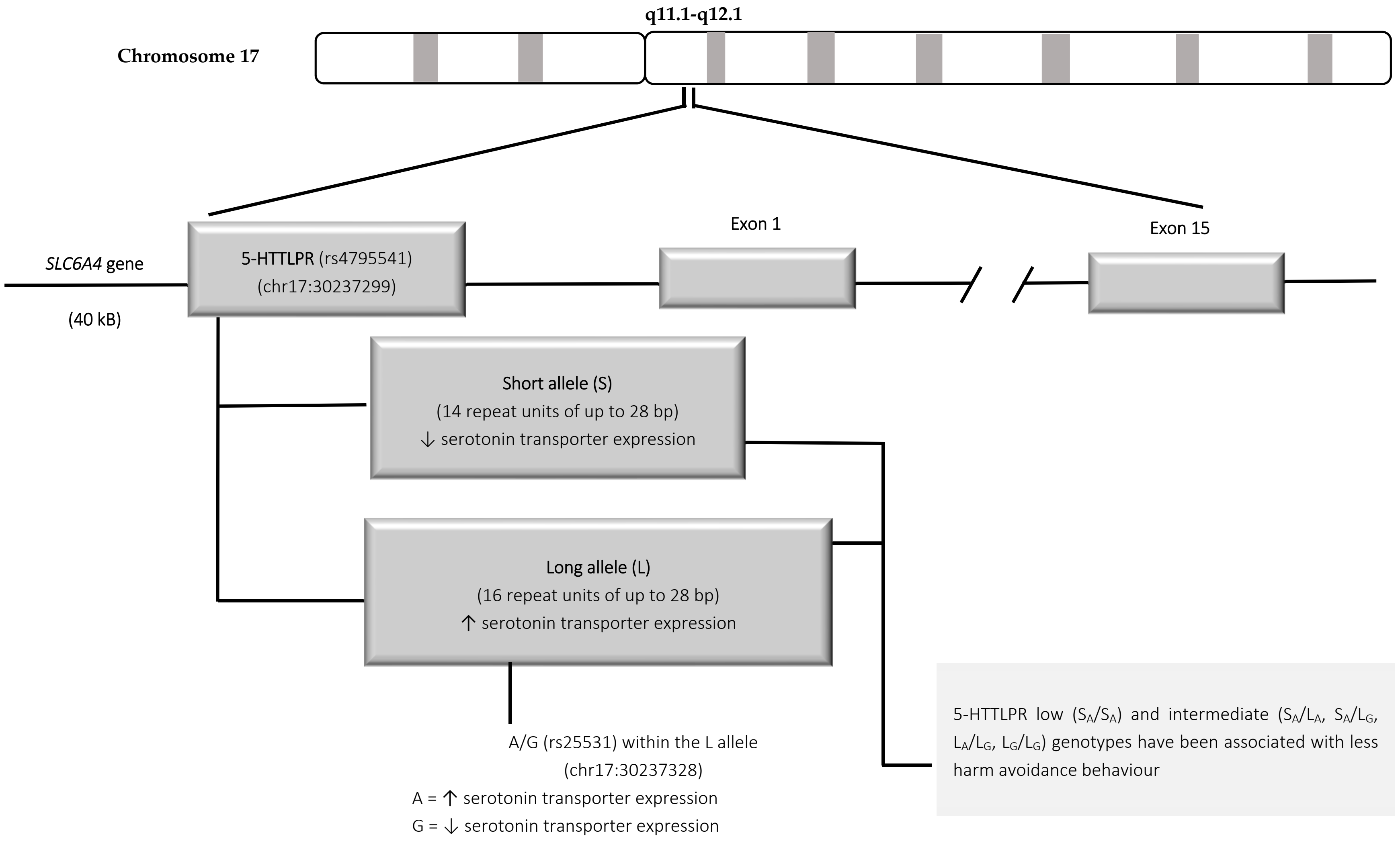

Figure S10. Schematic of rs4795541 5-HTTLPR, in conjunction with rs25531.

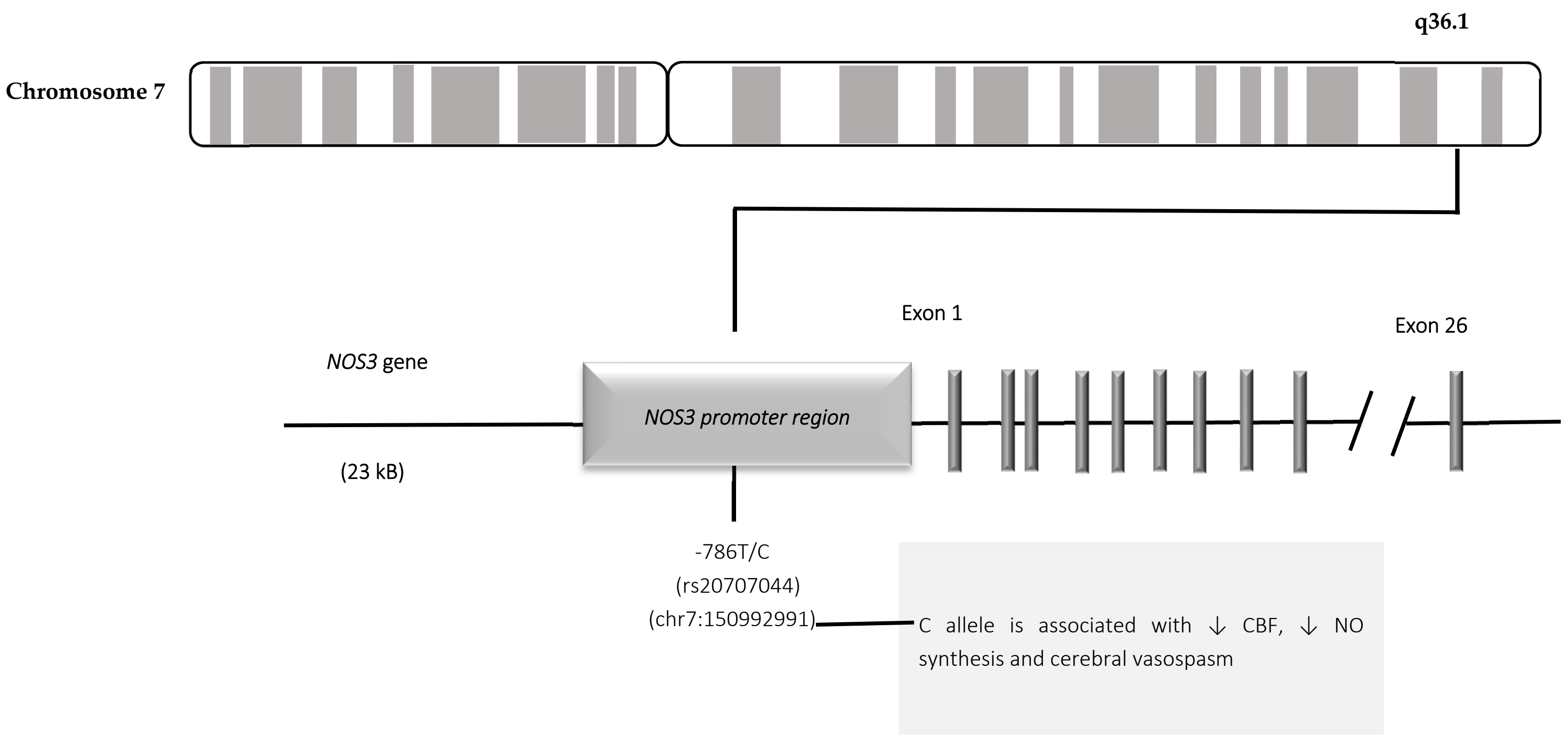

Figure S11. Schematic of rs2070744 *NOS3* C allele is associated with reduced cerebral blood flow in traumatic brain injury patients.

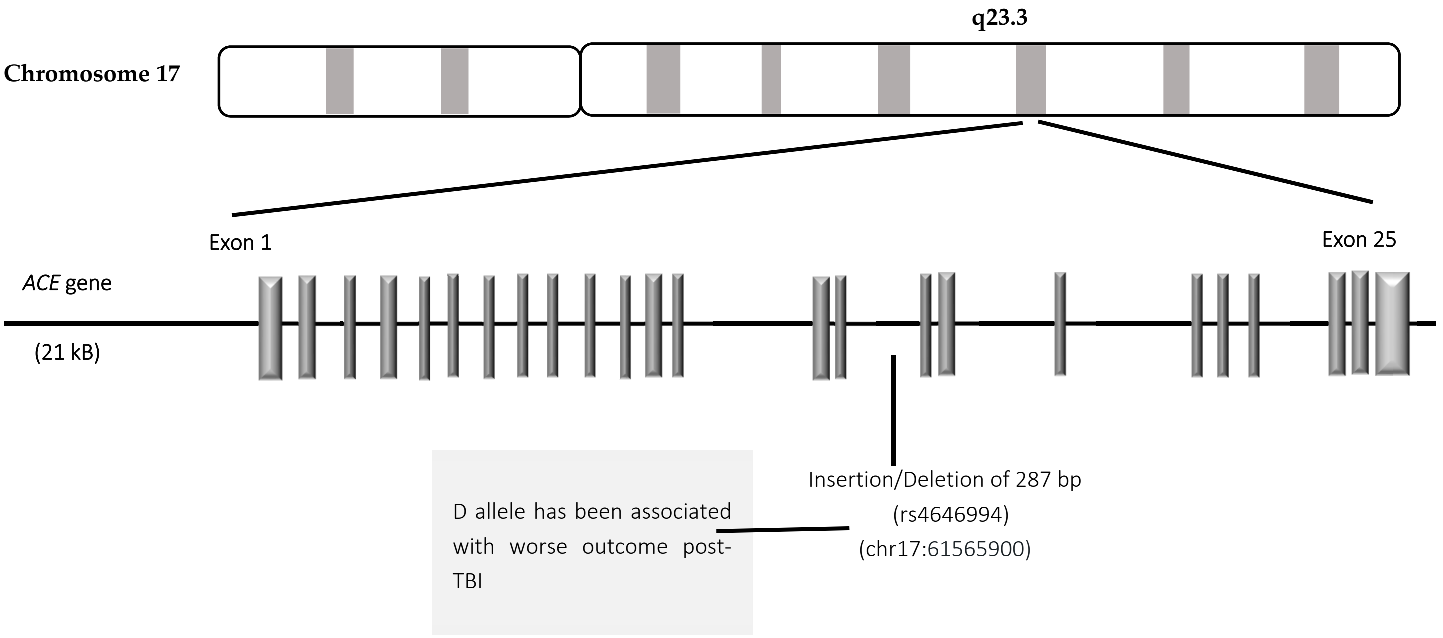

Figure S12. Schematic of *ACE* (rs4646994). A 287bp Alu insertion (I)/deletion (D) polymorphism, D/D homozygous carriers exhibit higher circulating and tissue ACE activity.

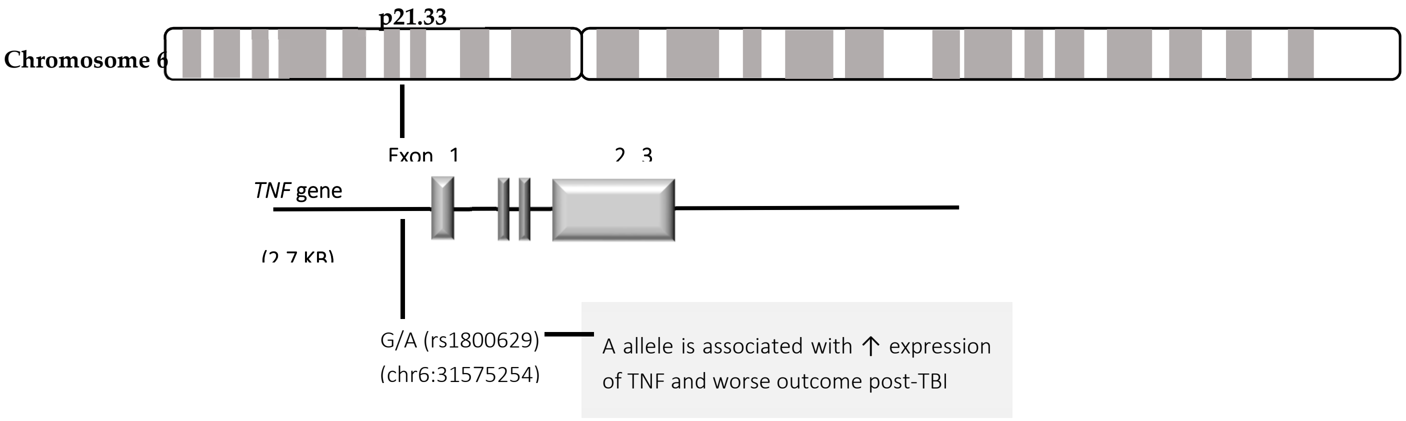

Figure S13. Schematic of *TNF* (rs1800629) A allele is associated with worse outcome post-TBI.

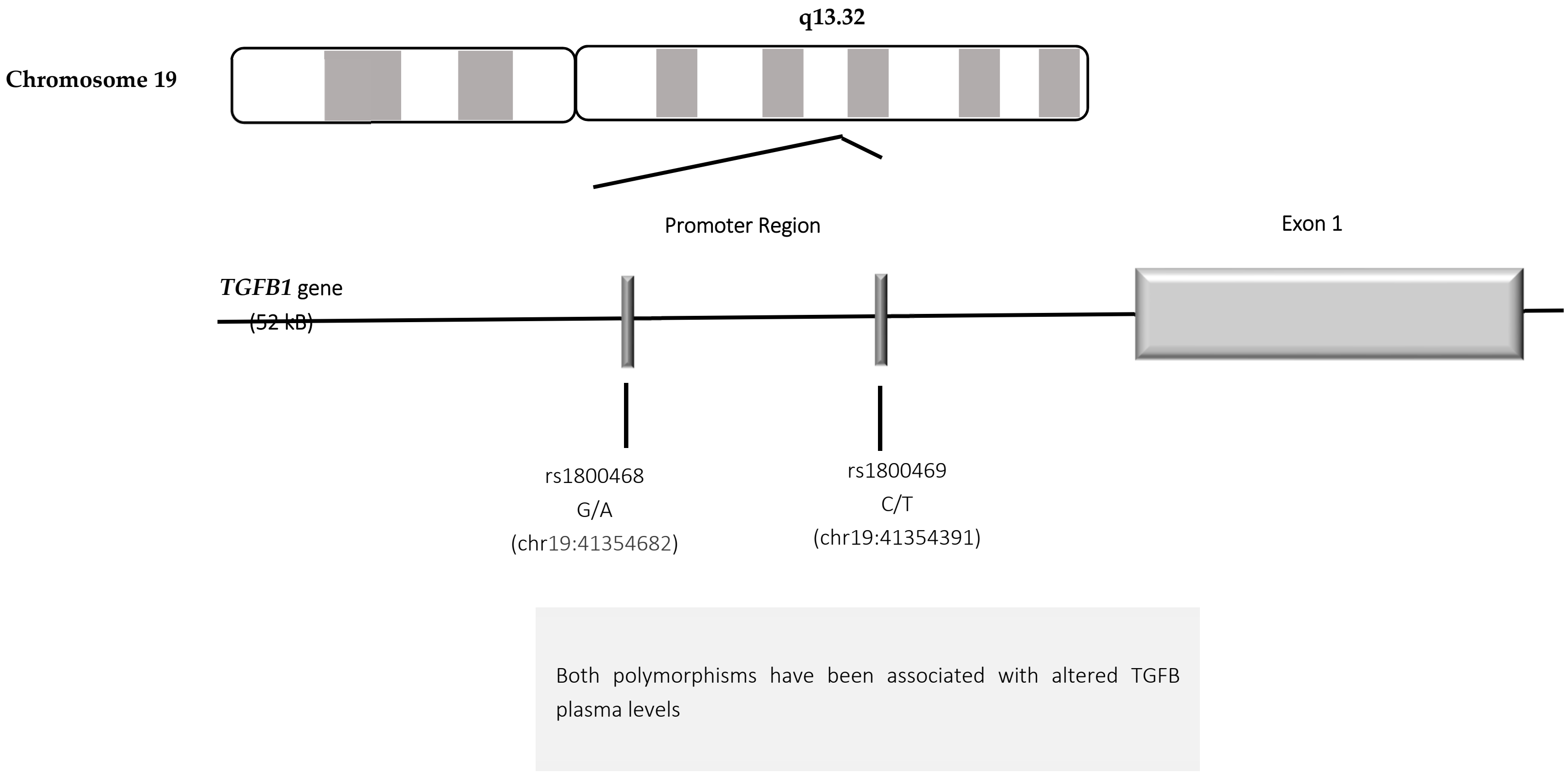

Figure S14. Schematic of *Transforming growth factor beta1* (rs1800468 and rs1800469).

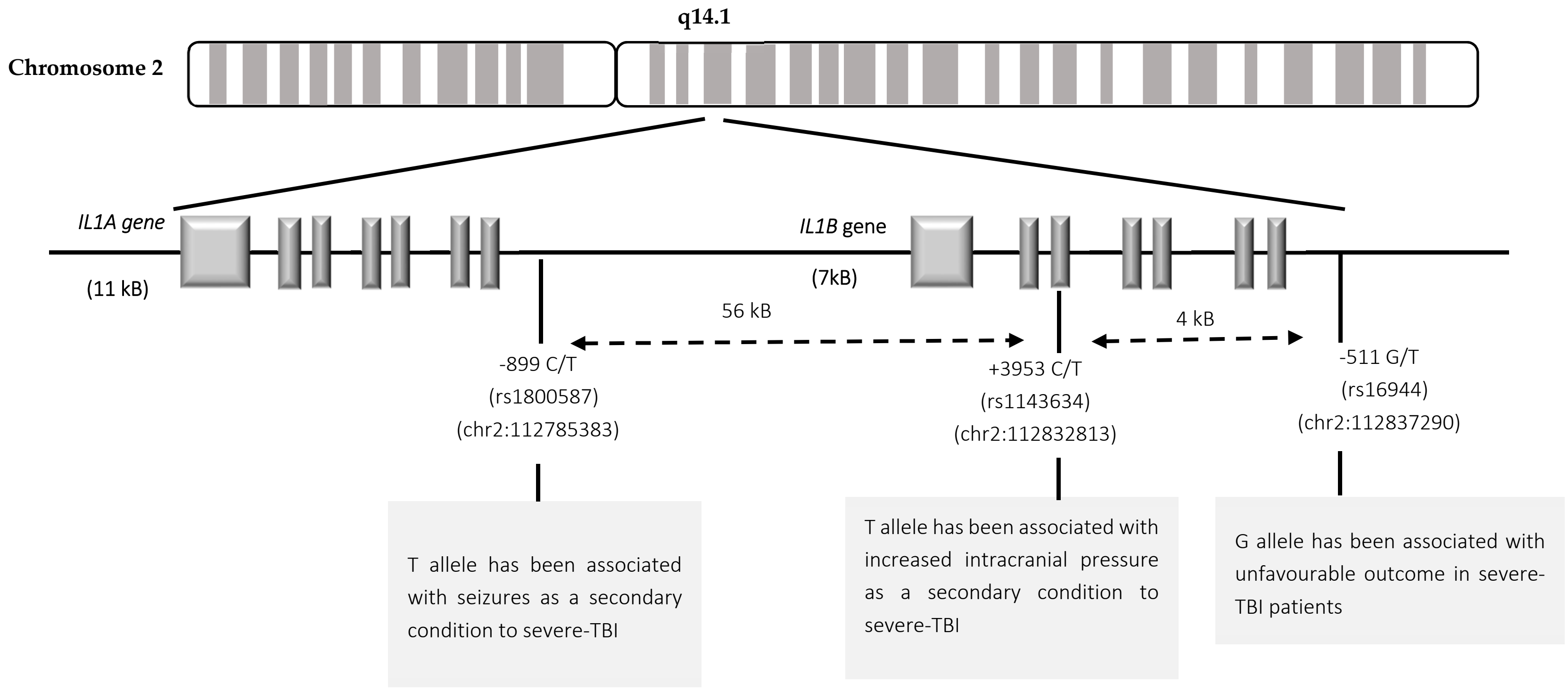

Figure S15. Schematic of *Interleukin 1 alpha* (rs1800587) *and Interleukin 1 beta* (rs16944 and rs1143634).

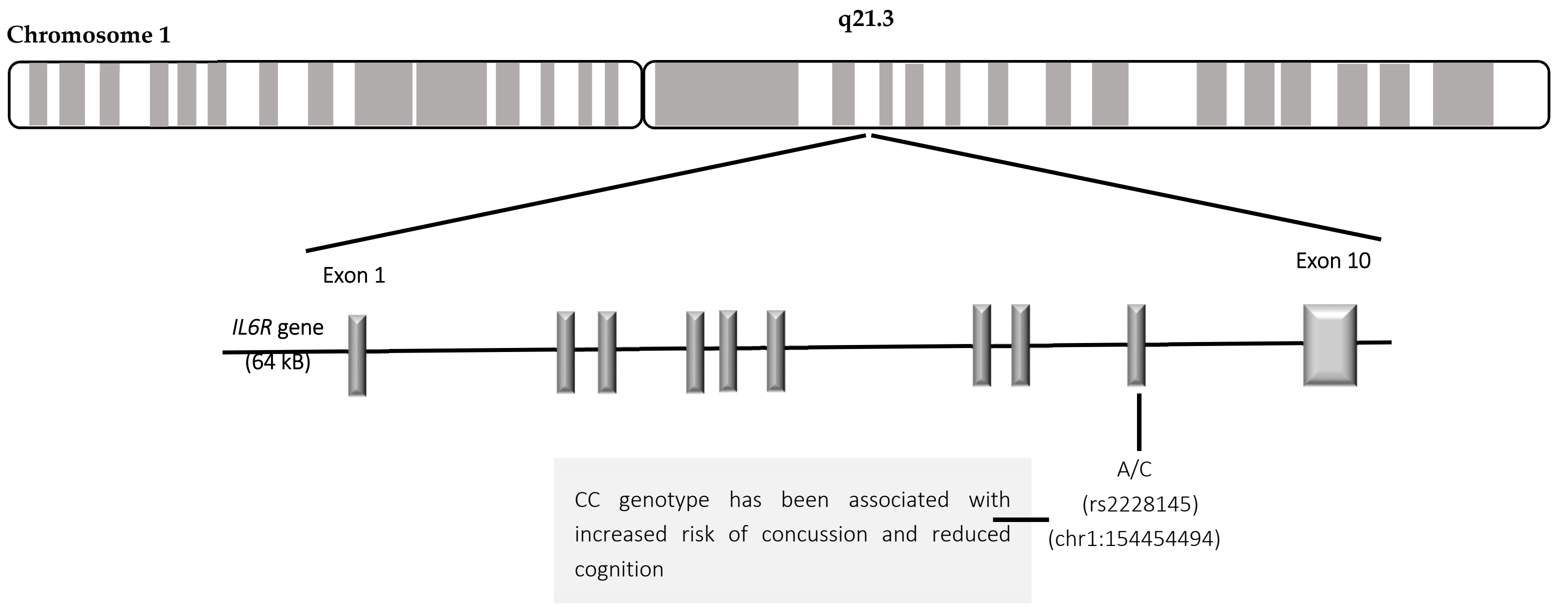

Figure S16. Schematic of rs2228145 *interleukin 6 receptor*. This SNP results in an amino acid substitution of aspartic acid for alanine and is associated with a two-fold increase in soluble IL6R serum levels.
